# The role of error risk taking and perceived organizational innovation climate in the relationship between perceived psychological safety and innovative work behavior: A moderated mediation model

**DOI:** 10.3389/fpsyg.2023.1042911

**Published:** 2023-01-19

**Authors:** Ahmed M. Elsayed, Bin Zhao, Abd El-mohsen Goda, Ahmed M. Elsetouhi

**Affiliations:** ^1^Business Administration Department, Faculty of Commerce, Mansoura University, Mansoura, Egypt; ^2^Beedie School of Business, Simon Fraser University, Burnaby, BC, Canada

**Keywords:** psychological safety, error risk taking, organizational innovation climate, innovative work behavior, individual level

## Abstract

To better understand how to motivate innovative work behavior (IWB) at the individual level in organizations, we investigate the link between perceived psychological safety and IWB and the role of error risk taking and perceived organizational innovation climate in this study. In particular, we hypothesize a moderated mediation model in which (a) perceived psychological safety is positively related to IWB, (b) error risk taking mediates the positive relationship between perceived psychological safety and IWB, and (c) perceived organizational innovation climate strengthens the positive link between error risk taking and IWB and the mediated link between perceived psychological safety and IWB *via* error risk taking. We tested the hypothesized model using data collected from 315 full-time employees working at six information and communication technology companies in a high-technology business district of Egypt. The findings largely support our hypotheses. We conclude by discussing the theoretical and practical implications.

## Introduction

Today’s organizations rely on innovation as a critical means to adapt to a business environment that is fast changing and highly competitive with demanding customer expectations (Widmann et al., 2016; Bos-Nehles et al., 2017; AlEssa and Durugbo, 2021). Organizations cannot innovate; people who work in organizations do (Sawyer, 2012): “One option for organizations to become more innovative is to encourage their employees to be innovative” (Agarwal, 2014; p. 43). Given the importance of understanding innovation in all its aspects, more research has been needed to examine how to encourage the innovative activities of employees (Bos-Nehles et al., 2017; Bos-Nehles and Veenendaal, 2019; AlEssa and Durugbo, 2021). In this study, we focus on innovative work behavior (IWB) of individual employees, which is defined as the intentional, self-initiated behavior of employees when they generate, introduce, and apply/implement new and useful ideas to enhance individual, team, and organizational performance (De Jong and Den Hartog, 2010; Bos-Nehles et al., 2017; AlEssa and Durugbo, 2021).

Besides personality predictors, many other individual and contextual factors affect IWB (see e.g., Ramamoorthy et al., 2005; Woods et al., 2017). We are interested in examining the role of perceived psychological safety in promoting IWB. Psychological safety perceptions in this study refer to perceptions of individual employees that the work environment is safe for taking interpersonal risks (Edmondson, 1999). Previous research has investigated the relationship between psychological safety and innovation, but mainly at the team and organizational levels (Edmondson and Lei, 2014; Frazier et al., 2017; Newman et al., 2017). To our best knowledge, little empirical work has been done at the individual level. Our study aims to fill this gap because, after all, organizational innovation originates from and relies on individual employees’ innovative activities (Sawyer, 2012). This study also addresses the recent call for more individual-level research on IWB to help us understand what factors change employees’ mentality from risk avoidance to risk taking for the purpose of building innovative work environments (AlEssa and Durugbo, 2021).

As a perceived psychological climate factor that fosters employees’ willingness to take risks in the workplace, psychological safety has been argued to promote risky behaviors such as innovation (Edmondson, 1999; Leung et al., 2015; Agarwal and Farndale, 2017; Newman et al., 2017). IWB involves employees’ breaking the status quo, challenging traditional working methods, and creating novel ideas (Shanker et al., 2017; Woods et al., 2017). By nature, IWB is a risky behavior because employees might have to face and manage resistance from peers and managers throughout the process, and, even after employees get the needed resources to implement their innovative ideas, employees face the risk of failure leading to reputation damage and even job loss. Therefore, we theorize and study the link between perceived psychological safety and IWB.

Furthermore, we aim to shed light on the causal mechanisms underlying this link by examining error risk taking as a mediator. Building on Rybowiak et al. (1999) conceptualization of error risk taking as a general attitude toward errors at work, we define error risk taking as an employee attitude, and in particular, employee readiness and behavioral tendency to make decisions and take actions to accomplish task goals despite the possibility that they might commit errors during the process. Innovation inherently involves exploration in uncertainty, which can result in many mistakes and errors (Jalonen, 2012; Lei et al., 2016). Therefore, employees need to be willing to risk making errors and mistakes in order to engage in IWB. To prevent people from the fear of making errors, it is important they feel that errors will not be held against them and that they will be given the benefit of the doubt (Edmondson et al., 2004). Thus, we expect that error risk taking plays a mediating role in the association between perceived psychological safety and IWB. In other words, we speculate that error risk taking transmits the effect of perceived psychological safety on IWB.

Moreover, we argue that organizational innovation climate perceptions, as a key contingency factor, are crucial to ensure that error risk taking results in IWB. Perceived organizational innovation climate refers to perceptions of individual employees of the degree to which an organization’s policies and practices support and encourage employees’ innovation initiative and effort (Shanker et al., 2017; Newman et al., 2020). Findings from previous studies have consistently shown that organizational innovation climate is a critical factor encouraging employee innovation (Shanker et al., 2017; Kruft et al., 2018; Sönmez and Yıldırım, 2018; Zuraik and Kelly, 2019; Newman et al., 2020). Organizational innovation climate has been examined as a moderator in many studies on innovation (e.g., Oke et al., 2013; Khalili, 2016; Newman et al., 2020). We examine perceptions of organizational innovation climate as a moderator in the link between error risk taking and IWB.

We intend this study to make significant contributions in at least two ways to the existing literature. First, as explained earlier, it offers insights into the underlying mechanism through which perceived psychological safety is related to IWB at the individual level by examining the mediating role of error risk taking. The error literature has suggested that employees’ attitudes and behavioral reactions toward errors can be significantly influenced by the particular context in which they work (e.g., Edmondson, 1999; Zhao and Olivera, 2006; Zhao, 2011; Zhao et al., 2018; Emby et al., 2019). Error risk taking, as an error coping attitude, is subject to the influences of immediate organizational factors and thus mediates the effects of these situational factors on behavioral responses (Spielberger, 1972; Rybowiak et al., 1999; Rausch et al., 2017). Our findings contribute refined knowledge regarding the role of error risk taking in transmitting the effect of perceived psychological safety on IWB and advance our understanding of individual factors that predict IWB. Second, this research also contributes to the employee innovation and creativity literature by examining the moderating role of perceived organizational innovation climate in the direct link between error risk taking and IWB and also in the indirect link between perceived psychological safety and IWB *via* error risk taking. Our study discusses and highlights the relevance and importance of perceptions of this organizational climate factor as a key contingency factor in relating perceived psychological safety and employee error risk taking to employee innovation.

This paper is organized as follows: The next section describes the theoretical background and rationale for the hypotheses. The method and results sections of the paper present details about the study sample, the measures used in the study, the data analyses performed, and the main findings. This is followed by the discussion section, which presents the implications for management theory and practice, the limitations of this study, and the directions for future research.

## Theoretical background and hypotheses

Prior research has found that psychological safety enhances innovation at the team (e.g., Burke et al., 2006; Post, 2012) and organizational (e.g., Baer and Frese, 2003; Edmondson and Lei, 2014) levels, primarily by facilitating information sharing and learning. At the individual level, although a positive link between psychological safety and employee IWB has been suggested and supported in the literature (e.g., Edmondson and Lei, 2014; Newman et al., 2017), little empirical research has been done to reveal the psychological mechanisms underlying the link. This is understandable, as psychological safety was originally grounded in the organizational learning literature. As a result, early empirical work has mainly focused on understanding the relationship between psychological safety and team- and organizational-level outcomes.

**FIGURE 1:**
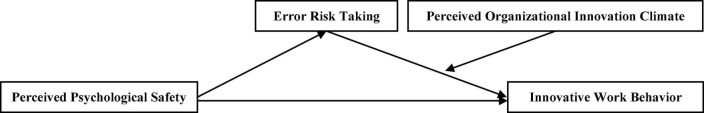
Hypothesized model.

We aim to fill this gap. In addition to studying the link between perceived psychological safety and IWB, we also examine error risk taking as a mediator in the link between perceived psychological safety and IWB. Furthermore, we study perceptions of organizational innovation climate as a contingency factor and reveal the moderator effect of this key factor in the link between error risk taking and IWB and the mediated link between perceived psychological safety and IWB *via* error risk taking. The hypothesized model is summarized in Figure 1.

### Perceived psychological safety and innovative work behavior

Innovative work behavior (IWB) is a non-routine behavior of employees that challenges the conventional way of doing things by presenting novel and different perspectives on how work is supposed to be done (Shanker et al., 2017; Woods et al., 2017; AlEssa and Durugbo, 2021). IWB is inherently risky because it challenges the status quo. IWB includes four key component activities: idea exploration, generation, championing, and implementation (De Jong and Den Hartog, 2010). Employees taking part in any combination of these activities are considered participating in IWB (Scott and Bruce, 1994). Feeling psychologically safe helps reduce perceived interpersonal risks and encourages employees to engage in all the four component activities.

Exploring and generating novel ideas involve working with a high level of uncertainty and ambiguity. Employees need to critically examine current products, services, or processes to come up with alternative ways to improve them. When employees feel psychologically safe, they have the needed level of energy, enthusiasm, and spirit to overcome anxiety associated with exploring in great uncertainty and ambiguity (Kark and Carmeli, 2009). Despite uncertainty about the success of their efforts, psychologically safe employees feel motivated to commit time and effort to exploring in different directions. As a result, employees are more likely to be successful in novel idea generation, which involves information search, combination, and reorganization beyond existing concepts (De Jong and Den Hartog, 2010).

Once creative ideas are generated, employees need to engage in idea championing to increase the likelihood of the acceptance and realization of these ideas. Idea championing requires employees to actively and enthusiastically promote the novel ideas, which could be taken as foolish, unrealistic, or unachievable by others; oftentimes employees may also have to overcome resistance to changes from all parties involved (Kark and Carmeli, 2009; De Jong and Den Hartog, 2010). Psychologically safe employees do not worry about potential negative interpersonal consequences and are comfortable with voicing different perspectives and speaking up freely to propose novel ideas (Edmondson and Lei, 2014). Also, they tend not to get defensive and are good at seeking and handling feedback (Schein, 1993; Carmeli et al., 2010; Javed et al., 2017). As a result, perceived psychological safety allows employees to access the needed psychological and social resources necessary for promoting novel ideas by helping employees overcome social rejection anxiety (Agarwal and Farndale, 2017).

If employees fail to implement their novel ideas and deliver their plans successfully, they put their careers at risk (De Jong et al., 2011). Perceived psychological safety promotes IWB by reducing perceived risks and costs associated with innovation failure (Leung et al., 2015; Sun and Huang, 2020). Furthermore, in order to implement innovative ideas, employees need to proactively seek help, support, and resources. Perceived psychological safety has been found to enhance the likelihood that employees successfully acquire approval and resources needed for implementing innovative ideas and transforming them into useful applications (e.g., Javed et al., 2017). Combining the aforementioned arguments, we hypothesize as follows:

***H1: Perceived psychological safety is positively related to innovative work behavior.***

### Perceived psychological safety and error risk taking

As mentioned earlier in the Introduction, error risk taking refers to employee openness toward error occurrence and readiness to make decisions or take actions to achieve task goals with the clear realization that errors and mistakes might be made during the process (Rybowiak et al., 1999; Tjosvold and Yu, 2007; Farnese et al., 2020). Following theorization of discretionary and risky behavior in organizations (e.g., Morrison and Phelps, 1999), we posit that error risk taking is based on a cost-benefit situational appraisal: individual employees are willing to take error-related risks when they believe that there are more benefits (e.g., achieving a desired task goal such as innovation through trial and error) than costs (i.e., negative consequences of errors, such as impaired personal image and interpersonal relationships). Arguing in this vein, perceived psychological safety is positively related to error risk taking through two mechanisms.

First, perceived psychological safety promotes error risk taking by lowering perceived negative consequences of error risk taking. Working in an organization where psychological safety is absent, employees are hesitant to risk making errors because they fear of all the potential, negative consequences associated with committing errors at work (Edmondson, 1996; Edmondson and Lei, 2014). The more severe the negative consequences are perceived to be, the more conservative and rigid employees become. In contrast, if employees feel psychologically safe, they perceive few negative consequences of errors and believe that no one will hold their errors against them. For example, one of the negative consequences of errors is damage to personal image (Zhao and Olivera, 2006). Perceived psychological safety minimizes such concerns by encouraging all employees to be themselves, not having to worry about any negative effect on their self-image, status, or career when committing errors (Kahn, 1990; Edmondson, 2002). Additionally, perceived psychological safety makes employees feel that interpersonal relationships will not be impaired in any way when they make mistakes at work; employees will not be blamed or rejected by coworkers or lose the support of the group or organization for admitting errors (Edmondson, 1999; Cannon and Edmondson, 2001; Gong et al., 2012).

Second, perceived psychological safety encourages employees to take error risks by increasing the perceived benefits of error risk taking. In a psychologically safe workplace, employees can expect dependability, structure, and clarity in the task environment and thus have more confidence that they will be able to get needed feedback and assistance from coworkers in order to learn from errors (Joseph, 2016). They do not have to fear humiliation by or resistance from others when they openly discuss their errors. Instead, given trusting and respectful interpersonal relationships, employees can expect open information exchange and support for reflective learning activities (Carmeli, 2007; Carmeli and Gittell, 2009; Gong et al., 2012; Edmondson and Lei, 2014). Without the need to defend and protect themselves, employees perceive a higher likelihood of success from their error risk-taking activities because they can fully concentrate on task-related activities such as error-based learning and problem solving (Kahn, 1990).

To conclude, perceived psychological safety makes it possible for employees to perceive more benefits than costs associated with error risk taking. As a result, employees remain open, flexible, and willing to take the risk to err.

***H2: Perceived psychological safety is positively related to error risk taking.***

### Error risk taking and innovative work behavior

Innovation is a process fraught with uncertainty in which one submits to the unknown and might commit mistakes and errors in the process (Jalonen, 2012). Therefore, errors are expected in the development of innovative products, processes, or service (De Jong and Den Hartog, 2010; Frese and Keith, 2015). As innovation is a process of trial and error (Ortt and Smits, 2006), employees who are open to taking error-related risks are more likely to engage in IWB.

We hypothesize that there is a positive link between error risk taking and IWB. Error risk takers are willing and motivated to participate in all four component activities of innovation. For example, employees are more open to experiment if they are willing to take risks to err (Rybowiak et al., 1999). If they err, error risk takers believe in the informative value of errors (Farnese et al., 2020) and are more likely to turn lessons learned from errors into novel ideas [idea exploration and generation (Frese and Keith, 2015)]. Also, because error risk takers feel comfortable with taking the responsibility to face and handle all the failures and errors head on (Rybowiak et al., 1999), they have enough courage and energy to engage in idea championing and implementation. Given the adaptability and flexibility associated with error risk taking (Rybowiak et al., 1999; Farnese et al., 2020), even if these employees fail in their championing or implementation initiatives, they are quick at learning and recovering from errors because they do not get defensive easily and do tend to stay task focused (Arenas et al., 2006; Tjosvold and Yu, 2007). They have a positive mindset toward errors in general and view them as valuable learning opportunities (Frese and Keith, 2015; Farnese et al., 2020). Therefore, they are astute at taking in different perspectives and fixing problems, increasing their likelihood of success in idea championing and implementation. To conclude, employees who are willing to take error-related risks are more likely to develop innovative solutions at work and to exhibit IWB.

***H3: Error risk taking is positively related to innovative work behavior.***

### Mediating role of error risk taking

Combining H2 and H3, we further posit that error risk taking mediates the relationship between perceived psychological safety and IWB. In other words, we speculate that error risk taking transits the effect of psychological safety on IWB. Although perceived psychological safety is positively related to IWB primarily by decreasing perceived risks in all four component activities involved in IWB, perceptions of psychological safety can also encourage IWB by dampening threat perceptions of errors and enhancing employees’ willingness to take error-related risks. Simply feeling psychologically safe does not guarantee that employees will engage in IWB. Employees’ involvement in IWB also depends on whether they have the right attitude and mindset toward errors (i.e., viewing errors as challenges as opposed to threats), which enables them to have the courage to innovate without fear of committing errors (Edmondson, 1999; Frese and Keith, 2015) and to have what it takes to convert errors into innovation if they do err (e.g., Leung et al., 2015; Newman et al., 2017). To conclude, we hypothesize that error risk taking mediates the effect of perceived psychological safety on IWB.

***H4: Error risk taking mediates the relationship between perceived psychological safety and innovative work behavior.***

### Moderating role of perceived organizational innovation climate

Organizational climate has long been studied as a moderator when examining desired employee behaviors because it sends contextual cues to employees about expected behaviors in a particular organizational context (e.g., Ostroff et al., 2012). A highly relevant climate factor supporting IWB is organizational innovation climate (e.g., Parzefall et al., 2008; Kang et al., 2016; Afsar et al., 2017; Newman et al., 2020). In this study, we examine the moderating role of perceived organizational innovation climate, which reflects the extent to which employees believe that organizational policies and practices support employee innovation activities (Khalili, 2016; Shanker et al., 2017; Newman et al., 2020).

We hypothesize that perceived organizational innovation climate, as a key contingency factor, augments the positive association between error risk taking and IWB. When the level of perceived innovation climate is high, employee error risk-taking attitudes and openness toward error occurrence are more likely to lead to IWB given the perceived appreciation and resource support for innovation. Employees feel more motivated to engage in IWB because they are optimistic about their likelihood of success when they engage in all four component activities of innovation (Scott and Bruce, 1994). For example, given the perceived organizational support for innovation, employees would feel that innovation is expected, supported, and rewarded in the organization. As a result, employees with higher innovation climate perceptions would be more willing to actively engage in *idea exploration and generation*: using errors as opportunities to retrieve useful information and creative ideas (Kang et al., 2016; Zuraik and Kelly, 2019). Moreover, if employees believe there is adequate resource supply supporting innovation, they would feel confident about their success in *idea championing or implementation*. They would perceive less resistance but more collaboration for promoting, advancing, and implementing their novel ideas (Parzefall et al., 2008; Zuraik and Kelly, 2019). In contrast, employees with lower innovation climate perceptions would have pessimistic expectations about the success of their innovative initiatives (Yuan and Woodman, 2010). If employees do not think that their organization supports innovation, they will be reluctant to share the innovative ideas they have acquired from their error risk-taking activities. They will keep innovative ideas to themselves because they fear premature censure of their ideas. They will also refrain from implementing these ideas because they lack certainty that they have the needed time, support, and resource to bring the ideas to fruition (Khalili, 2016).

In conclusion, we posit that the positive link between error risk taking and IWB is stronger when employees have high rather than low perceptions of organizational innovation climate.

*H5: Perceived organizational innovation climate moderates the relationship between error risk taking and innovative work behavior such that the relationship will be more positive for employees with higher innovation climate perceptions.*

Furthermore, we expect that the indirect relationship between perceived psychological safety and IWB *via* error risk taking is moderated by perceived organizational innovation climate. Compared with low innovation climate, high innovation climate further reduces the perceived risks and uncertainties (e.g., Parzefall et al., 2008; Afsar and Umrani, 2020) that psychologically safe employees experience when they convert what they have learned from error risk taking to IWB. Accordingly, we hypothesize as follows:

*H6: Perceived organizational innovation climate moderates the mediated relationship between perceived psychological safety and innovative work behavior via error risk taking such that the relationship will be more positive for employees with higher innovation climate perceptions.*

## Materials and methods

### Participants and data collection procedures

Data were collected from full-time employees working in the information and communication technology companies in the Smart Village, Egypt. The Smart Village was established in 2001 to be the nucleus for building and growing the information technology industry in the country. Currently, it is the largest gated high-technology business community in Egypt.

We collected data using paper-and-pencil questionnaires. With the support and agreement of the top management, the human resources department in each company helped us distribute the study announcement, along with a letter assuring confidentiality and inviting voluntary participation among all their employees. Participants completed all the questionnaire sections in an office within their company. The principal investigator collected all the completed questionnaires sealed in an envelope on site to protect data confidentiality. After completing the survey, participants were thanked for their participation. They were given the principal investigator’s contact information in case they wanted more information regarding the study or needed to discuss their experience of participating in the study.

A total of 400 questionnaires were distributed, of which 386 were returned. After eliminating 7 questionnaires with missing responses and 64 responses from companies with less than 10 responses per company, the final sample comprised 315 valid responses from 6 companies (company age ranging from 20 to 34 years, mean = 20.66 years, S.D. = 6.42). Of the 315 respondents, 46.67 % were females; more than 75% were under 35 years old. As to the educational level, 86.35% were university graduates, 11.75% had a master’s degree, and 1.90% had a Ph.D. degree. Fifty-nine percent of respondents had work experience of more than 5 years, while 60.63% spent less than 5 years in the current position. For detailed demographic information, see Table 1.

**TABLE 1:** Control variables.

Variable	*n*	%
**Gender:**		
Male	168	53.33
Female	147	46.67
**Age:**		
Less than 25	81	25.71
From 25 to less than 30	82	26.03
From 30 to less than 35	82	26.03
From 35 to less than 40	37	11.75
From 40 to less than 45	16	5.08
More than 45	17	5.40
**Education:**		
University level	272	86.35
Master	37	11.75
Ph.D.	6	1.90
**Work experience:**		
Less than 5 years	127	40.32
From 5 to less than 10	99	31.43
From 10 to less than 15	57	18.10
From 15 to less than 20	20	6.34
More than 20	12	3.81
**Current job experience:**		
Less than 5 years	191	60.63
From 5 to less than 10	102	32.38
From 10 to less than 15	12	3.81
From 15 to less than 20	9	2.86
More than 20	1	.32

*N* = 315.

### Measures

Preexisting scales with established validity and reliability were used to measure the study variables. We followed the translation/back-translation procedure (Brislin, 1980; Behling and Law, 2000) to translate the scales from English to Arabic. To verify that the translated scale items reflected the constructs we intended to measure, a panel of five experts in human recourses management and organizational behavior was used to assess the content validity of the scales. We further modified the wording of the scale items upon the feedback from the panel. To assess and confirm the face validity of the scales, we invited five human recourses managers and ten employees from the information and communications technologies companies (i.e., from the target population) in the Smart Village to review all the scale items. Unless stated otherwise, all the measures used a 5-point Likert-type scale ranging from “strongly disagree” (1) to “strongly agree” (5).

Perceived psychological safety. Employee perception of psychological safety within the organization was measured using a 7-item scale developed by Edmondson (1999) and modified by Carmeli (2007). A sample item is “If you make a mistake in this organization, it is often held against you.” The Cronbach’s alpha for the scale was 0.96.

Error risk taking was measured using the 4-item scale from Rybowiak et al. (1999). In the scale instruction, we asked participants to focus on their current job and organization. A sample item follows: “If one wants to achieve at work, one has to risk making mistakes.” The Cronbach’s alpha was 0.95.

Perceived organizational innovation climate was assessed using six items adapted from Scott and Bruce (1994), evaluating individual employees’ perceptions regarding support for innovation from their current employer. Three items capture perceived organizational support for innovation (more of a supportive climate), and three items capture the degree to which the resource supply was perceived as adequate for pursuing innovation in the organization. Sample items include “This place seems to be more concerned with the status quo than with change (reverse-coded)” and “There are adequate resources devoted to innovation in this organization.” The Cronbach’s alpha was 0.88.

Innovative work behavior was measured using six items adapted from De Jong and Den Hartog (2010) on a 5-point Likert-type scale ranging from “Rarely” (1) to “Always” (5). A sample item is “In your job, how often do you make suggestions to improve current products or services?” The Cronbach’s alpha was 0.94.

Control variables. Empirical studies have shown that gender, age, education, work experience, and current job experience affect error risk taking (Fay and Frese, 2000; Yan et al., 2014; King and Beehr, 2017) as well as innovative work behavior (Scott and Bruce, 1994; Yuan and Woodman, 2010; Hapsari et al., 2019; Bibi and Afsar, 2020). Thus, we controlled for these variables in hypothesis testing. To account for the nested structure of our data, we included firm dummies to control for between-firm effects (Pustejovsky and Tipton, 2018; McNeish and Kelley, 2019).

### Measurement model

Before testing the proposed hypotheses, we conducted confirmatory factor analysis in AMOS to evaluate the factorial validity of all the measures. The hypothesized four-factor model (consisting of perceived psychological safety, error risk taking, perceived organizational innovation climate, and IWB) demonstrates a good fit to the data (χ^2^ = 576.67, df = 222, χ^2^/df = 2.60, CFI = 0.95, TLI = 0.95, NFI = 0.93, RMSEA = 0.07, SRMR = 0.06) (Hair et al., 2014; Thakkar, 2020). All the alternative modes fit significantly worse than the hypothesized model (comparison results are presented in Table 2).

**TABLE 2:** Confirmatory factor analysis.

Measurement models	χ^2^	df	χ^2^/df	CFI	TLI	NFI	SRMR	RMSEA	Δχ^2^ (Δdf)
Four-factor model	576.67	222	2.60	0.95	0.95	0.93	0.06	0.07	
Three-factor model	1739.06	227	7.66	0.81	0.78	0.78	0.21	0.15	1162.38*** (5)
Two-factor model	2901.61	229	12.67	0.66	0.62	0.64	0.16	0.19	2324.94*** (7)
Single-factor model	3316.94	230	14.42	0.60	0.56	0.59	0.18	0.21	2740.27*** (8)

*N* = 315. Four-factor model: psychological safety, error risk taking, innovation climate, IWB. Three-factor model: psychological safety, error risk taking, innovation climate + IWB. Two-factor model: psychological safety, error risk taking + innovation climate + IWB. Single-factor model: psychological safety + error risk taking + innovative climate + IWB. ***p < 0.001.

We also assessed scale reliability and validity. As shown in Table 3, all the scale factor loadings exceeded 0.5, showing acceptable reliability (Hair et al., 2014). Internal consistency reliability was assessed using Cronbach’s alpha (α) and the composite reliability (CR). Both Cronbach’s alpha and CR values were above the threshold value of 0.7 (Hair et al., 2014), suggesting that all the scales have sufficient internal consistency reliability. Convergent validity was measured by the average variance extracted (AVE); all AVEs were above 0.5, suggesting adequate convergent validity (Fornell and Larcker, 1981; Hair et al., 2014). Discriminant validity was evaluated using the square root of AVE, which was higher than the correlation between a focal construct and the other constructs, demonstrating discriminant validity for all the scales (Fornell and Larcker, 1981; Hair et al., 2014). To summarize, all the scales showed satisfactory reliability and validity.

**TABLE 3:** Scale reliability and validity.

Construct		Indicators		Individual loadings	Cronbach’s alpha	CR	AVE
Psychological safety		PS1		0.96	0.96	0.96	0.80
		PS2		0.94			
		PS3		0.95			
		PS4		0.56			
		PS5		0.94			
		PS6		0.94			
		PS7		0.88			
Error risk taking		ERT1		0.87	0.95	0.95	0.82
		ERT2		0.91			
		ERT3		0.93			
		ERT4		0.92			
Innovation climate	0.96	Support for innovation	SI1	0.84	0.88	0.84	0.73
			SI2	0.93			
			SI3	0.87			
	0.75	Resource supply	RS1	0.64			
			RS2	0.81			
			RS3	0.72			
Innovative work behavior		IWB1		0.88	0.94	0.94	0.73
		IWB2		0.91			
		IWB3		0.79			
		IWB4		0.86			
		IWB5		0.81			
		IWB6		0.88			

### Common method bias (CMB)

Egypt’s Central Agency for Public Mobilization and Statistics allowed us only to collect data from the employees in the participating companies in a one-time manner (cross-sectional data). Therefore, common method bias (CMB) is a potential problem in our data. Harman’s single-factor test was utilized to investigate potential CMB among the study variables (Podsakoff et al., 2003). The unrotated principal-component factor analysis extracted four factors with eigenvalues greater than 1; the first factor accounted for only 49% of the total variance. This result suggests that CMB is not likely to be a severe problem with our data. Also, given the low to modest level of responses for all the four key constructs (as shown in Table 4), social desirability bias is not a concrete concern in our data, either.

**TABLE 4:** Descriptive statistics and correlations.

	Mean	SD	1	2	3	4	5	6	7	8	9
1. Gender	0.47	0.50	-								
2. Age	2.61	1.40	-0.00	-							
3. Education	1.16	0.41	0.00	0.25**	-						
4. Work experience	2.02	1.09	-0.05	0.86**	0.16**	-					
5. Current job experience	1.50	0.73	-0.10	0.56**	0.11*	0.62**	-				
6. Perceived psychological safety	2.78	1.07	0.23**	0.20**	0.03	0.18**	0.10*	**(0**.**89)**			
7. Error risk taking	2.81	1.14	-0.03	0.37**	0.04	0.39**	0.16**	0.64***	**(0**.**91)**		
8. Perceived innovation climate	1.54	0.54	0.26**	0.16**	0.05	0.15**	0.10*	0.40***	0.40***	**(0**.**86)**	
9. Innovative work behavior	2.94	0.86	0.08	0.25**	0.10	0.28**	0.19**	0.85***	0.52***	0.41***	**(0**.**86)**

Square root of AVE is presented in the bold parentheses along the diagonal; *p < 0.05; **p < 0.01; ***p < 0.001.

## Results

Descriptive statistics, correlations, and reliability coefficients of the variables included in the study are presented in Table 4.

We tested our hypotheses in three steps. First, we tested Hypothesis 1 using a simple regression model. Second, we added the mediator (error risk taking) in the model, and tested the mediation hypothesis using SPSS macro PROCESS Model 4 (Hayes, 2022) with 1,000 bootstraps resamples. Finally, we utilized the SPSS macro PROCESS Model 14 (Preacher et al., 2007; Hayes, 2022) to test the overall moderated mediation model. In all the analyses, the control variables were gender, age, education, work experience, current job experience, and firm dummies.

As shown in Table 5, after including control variables in model testing, perceived psychological safety was positively related to IWB (β = 0.31, *p* < 0.001, 95% CI [0.23, 0.39]). Thus, Hypothesis 1 was supported. Data analysis results also revealed that perceived psychological safety was significantly related to error risk taking (the mediator) (β = 0.69, *p* < 0.001, 95% CI [0.61, 0.78]), and error risk taking was significantly related to IWB (β = 0.15, *p* < 0.001, 95% CI [0.04, 0.26]), providing support for Hypothesis 2 and Hypothesis 3.

**TABLE 5:** Model testing results.

Variables	Model 1			Model 2				Model 3	
	Innovative work behavior			Error risk taking		Innovative work behavior		Innovative work behavior	
	β (SE)	95% CI		β (SE)	95% CI	β (SE)	95% CI	β (SE)	95% CI
Constant	1.60 (0.18)***	[1.11, 2.00]		0.68 (0.23)**	[0.23, 1.13]	1.35 (0.23)***	[0.90, 1.79]	1.90 (0.42)***	[1.08, 2.72]
Gender	0.01 (0.09)	[−0.18, 0.18]		−0.42 (0.09)***	[−0.61, −0.24]	0.057 (0.095)	[−0.13, 0.24]	0.04 (0.09)	[−0.15, 0.23]
Age	−0.05 (0.06)	[−0.18, 0.10]		0.04 (0.07)	[−0.09, 0.17]	−0.047 (0.064)	[−0.17, 0.08]	−0.03 (0.06)	[−0.15, 0.10]
Education	0.12 (0.11)	[−0.11, 0.39]		−0.10 (0.11)	[−0.32, 0.12]	0.148 (0.111)	[−0.07, 0.37]	0.10 (0.11)	[−0.12, 0.32]
Work experience	0.20 (0.08)	[0.03, 0.37]		0.33 (0.09)***	[0.16, 0.50]	0.142 (0.087)	[−0.03, 0.31]	0.11 (0.09)	[−0.06, 0.28]
Current job experience	0.04 (0.08)	[−0.12, 0.19]		−0.23 (0.08)**	[−0.38, −0.08]	0.079 (0.077)	[−0.07, 0.23]	0.07 (0.08)	[−0.08, 0.22]
Perceived psychological safety (PS)	0.31 (0.04)***	[0.23, 0.39]		0.69 (0.04)***	[0.61, 0.78]	0.21 (0.06)***	[0.10, 0.32]	−0.06 (0.10)	[−0.25, 0.14]
Error risk taking (ERT)						0.15 (0.06)**	[0.04, 0.26]	−0.09 (0.14)	[−0.37, 0.18]
Perceived organizational innovation climate (IC)								0.15 (0.28)	[−0.39, 0.70]
IC × ERT								0.16 (0.08)^†^	[−0.00, 0.32]
R^2^		0.23			0.55		0.25		0.28
**Bootstrap results for indirect effect:**						**Value (BootSE)**	**95% CI**		
PS → ERT → IWB						0.10 (0.04)	[0.03, 0.19]		
**Indirect effect and significance using Sobel test:**						**Value (*SE*)**			
PS → ERT → IWB						2.64 (0.04)**			
**Conditional indirect effects PS → ERT → IWB:**								**Effect (*BootSE*)**	**95% CI**
Perceived organizational innovation climate (IC)			“Low” (−1 SD)					0.04 (0.05)	[−0.05, 0.15]
			“Moderate” (mean) IC					0.10 (0.04)	[0.02, 0.18]
			“High” (+1 SD) IC					0.16 (0.05)	[0.07, 0.26]
**Index of moderated mediation:**								**Index (*BootSE*)**	**95% CI**
Perceived organizational innovation climate (IC)								0.11 (0.05)	[0.00, 0.22]

^†^*p* < 0.1; **p < 0.01; ***p < 0.001.

To assess the significance of the mediation, we first examined the indirect effect based on the bootstrapped confidence intervals, which showed that the indirect effect of perceived psychological safety on innovative work behavior *via* error risk taking was significant (estimate = 0.10, 95% CI [0.03, 0.19]), providing support for Hypothesis 4. Second, we used the Sobel (1982) test (two-tailed), which confirmed that error risk taking mediated the relationship between perceived psychological safety and IWB (estimate = 2.64, *p* < 0.01). Together, these results support the mediation role of error risk taking in the link between perceived psychological safety and IWB. The testing results are presented in Table 5.

We next utilized the SPSS macro PROCESS Model 14 (Preacher et al., 2007; Hayes, 2022) to test whether the influence of error risk taking on IWB was moderated by perceived organizational innovation climate. In line with H5, we observe a marginally significant interaction effect of error risk taking and perceived organizational innovation climate (β = 0.16, *p* < 0.1, 95% CI [−0.00, 0.32]).

**FIGURE 2:**
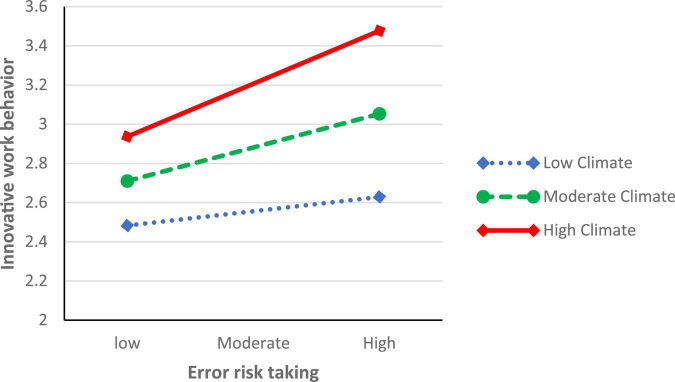
Interaction between perceived organizational innovation climate and error risk taking.

The hypothesized moderated mediation effect was assessed by checking whether the strength of the indirect effect was moderated by perceived organizational innovation climate (Preacher et al., 2007). As shown in Table 5, the indirect path between perceived psychological safety and IWB *via* error risk taking was stronger at the high level of perceived organizational innovation climate (estimate = 0.16, 95% CI [0.07, 0.26]) compared to the moderate level of perceived innovation climate (estimate = 0.10, 95% CI [0.02, 0.18]) and the low level of perceived innovation climate (estimate = 0.04, 95% CI [−0.05, 0.15]). Further, the index of moderated mediation, which indicates that “any two conditional indirect effects estimated at different values of the moderator are significantly different from one another” (Hayes, 2015, p. 2), was significant (Index = 0.11, 95% CI [0.00, 0.22]), supporting Hypothesis 6. Figure 2 illustrates the interaction between organizational innovation climate perceptions and error risk taking. The graph shows that, if the perception of organizational innovation climate is high, the conditional effect will be stronger such that the indirect effect between psychological safety and IWB *via* error risk taking will be augmented.

## Discussion

The main focus of the current research is to extend our understanding of the relationship between perceived psychological safety and innovative work behavior at the individual level by examining the role of error risk taking and perceived organizational innovation climate in a moderated mediation model. We started where the previous studies left off, which suggested that employees will engage in innovative activities when they perceive that it is safe to take interpersonal risks at work (Leung et al., 2015; Javed et al., 2017; Sun and Huang, 2020). Besides this positive link between perceived psychological safety and IWB suggested in the literature, we theorized and studied the mediating role of error risk taking in the link between perceived psychological safety and IWB. Our data analysis results support these hypothesized relationships. Hence, perceived psychological safety minimizes concerns individuals have in terms of interpersonal risks associated with IWB and also risks in making errors at work. Findings from this study also confirm the moderating role of the perceived organizational innovation climate in the indirect relationship between perceived psychological safety and IWB *via* error risk taking. We next discuss theoretical and practical implications of this study.

### Theoretical contributions

Our study contributes to the literature in several ways. First, this study responds to the call urging further work on promoting IWB by improving employee risk acceptance (Newman et al., 2020; AlEssa and Durugbo, 2021). This seems to be even more relevant and timely in this post-pandemic era, when employees may have generally become more risk averse given the sense of insecurity (together with a high level of perceived risks to one’s safety and health) experienced during the pandemic (e.g., Al-Thaqeb et al., 2022).

Second, our finding that perceived psychological safety is positively related to IWB confirms the important role played by psychological safety in minimizing perceived interpersonal risks (Edmondson and Lei, 2014; Frazier et al., 2017; Newman et al., 2017). More importantly, our finding regarding the mediating role played by error risk taking in the link between perceived psychological safety and IWB adds new insights to the literature, revealing that perceived psychological safety is instrumental in promoting IWB by reducing concerns employees might have about taking error-related risks in innovation activities. These findings not only confirm the positive link between psychological safety and employee IWB suggested in the literature (e.g., Edmondson and Lei, 2014; Newman et al., 2017) but also reveal the psychological mechanism underlying the link. While we know from prior research that psychological safety promotes innovation at the team (e.g., Burke et al., 2006; Post, 2012) and organizational (e.g., Baer and Frese, 2003; Edmondson and Lei, 2014) levels primarily by encouraging open information exchange and team/organizational learning, little empirical work has been done at the individual level to help us understand what mediators transmit the effects of psychological safety on employee IWB. Our theorization and findings regarding the mediating role of error risk taking fill this gap.

Third, our data support our conceptualization of error risk taking as an error coping attitude that is influenced by immediate situational factors and its important role in encouraging IWB. Although errors are expected in any innovative work (Frese and Keith, 2015; Lei et al., 2016), this is the first study to directly investigate the link between error risk taking and IWB, highlighting the importance of cultivating in employees the mental openness, the psychological preparedness, and the behavioral readiness for the occurrence of errors in organizations where innovation is valued.

Fourth, we contribute to the literature on innovation by confirming the moderating role of perceived organizational innovation climate, as a critical contingency factor, in the indirect link between perceived psychological safety and IWB *via* error risk taking. The current research not only confirms perceived organizational innovation climate as a key supportive mechanism in enhancing IWB (Newman et al., 2020) but also complements prior research by revealing the interplay between perceived psychological safety and organizational innovation climate perceptions. While feelings of being psychologically safe might lead to employee IWB by dampening threat perceptions and encouraging employees to take error-related risks, perceived organizational innovation climate strengthens this indirect positive link between psychological safety and valuable IWB by further reducing the risks and uncertainties employees might perceive when they engage in IWB (e.g., Parzefall et al., 2008; Afsar and Umrani, 2020). When perceived organizational innovation climate is high, employees perceive IWB as even less risky because they believe they will get the needed resource supply, support, and appreciation for IWB.

Last but not the least, our study reveals and confirms the important role played by perceived psychological safety and error risk taking in IWB in a relatively understudied cultural context: Egypt. Replication studies are needed in business and management research (Ryan and Tipu, 2022), validating the key concepts and the related findings in different cultural contexts to provide evidence of their generalizability.

### Practical implications

Findings from this study can help management understand and promote employee IWB. Our results highlight the role of perceived psychological safety in shaping employee attitudes toward error-related risks and promoting employee IWB. Working in a psychologically safe organization, employees are willing to risk making mistakes in order to come up with creative ideas for improving products, services, and processes, as opposed to worrying about all the risks involved and not trying anything new. In the post-pandemic era, management should proactively engage in efforts to provide employees with a psychologically safe work environment so as to encourage employee creativity and innovation. For example, for employee training and development, management should use error-management training instead of error-avoidance training (Keith and Frese, 2005; Tiwari and Lenka, 2016). In daily work, managers should learn how to deliver constructive feedback upon detection of employee errors and, in so doing, help employees develop a positive, learning-oriented mindset toward errors. To facilitate innovation, management can also organize meetings after error or failure detection to encourage employees to share their valuable lessons learned through reflections and analyses of errors or failure. Such open communication and targeted training can help build psychological safety by facilitating interpersonal trust and connections and an awareness of interdependence among employees (e.g., Dusenberry and Robinson, 2020).

Transmitting the effect of psychological safety, employee error risk taking was found to be positively related to IWB. This finding offers practical implications for organizations that value innovation and desire to further promote IWB among their employees. Error risk taking is indeed subject to the influences of immediate situation factors and can be promoted in organizations by creating a psychologically safe workplace. Also, to turn valuable lessons learned from error risk taking to innovation, perceived organizational innovation climate is an important facilitating factor (Newman et al., 2020). Managers who value IWB should carefully review and examine their organizational policies and practices to make sure that employees perceive both intangible support (e.g., organizational culture, value, and norms) and resource supply (e.g., time, money, technical assistance, and materials needed) for innovation from the organization. More importantly, to create and maintain a strong innovation climate, management should send clear and consistent signals to employees, emphasizing the organization’s support for innovation (e.g., Hogan and Coote, 2014).

### Limitations and future research directions

Our findings should be interpreted with caution due to the following limitations. First, we collected data from the information and communication technology companies in the Smart Village of Egypt. Future research is needed to test whether our findings hold across different industries and cultures. In particular, all the participants in this study had at least a university degree, were relatively young, and had relatively short work experience or current job experience, reflecting the demographic profile of the typical workforce of the Smart Village. It remains to see whether our findings apply to populations with different education, age, and work experience profiles.

Second, due to the limit imposed by the government agency in Egypt, we had to use a cross-sectional design to collect data, which weakens our confidence in the causal relationships tested. Also, we used self-reported data for all the variables. Although we found no evidence of common method bias, we encourage future research to use time-lagged, multi-source methods to collect data to confirm the robustness of our tested linkages.

Finally, our data were collected during the COVID-19 pandemic, which might have led to a lower-than-usual level of responses for error risk taking, as employees might have felt risk averse during the pandemic because they perceived a relatively high level of health risks on a daily basis caused by the spread of a highly contagious and dangerous disease (Al-Thaqeb et al., 2022). The responses for psychological safety could be lower than normal for the same reason. Therefore, our findings should be interpreted by keeping this limitation in mind.

## Data availability statement

The raw data supporting the conclusions of this article will be made available by the authors, without undue reservation.

## Ethics statement

The studies involving human participants were reviewed and approved by the Central Agency for Public Mobilization and Statistics, Egypt. Written informed consent for participation was not required for this study in accordance with the national legislation and the institutional requirements.

## Author contributions

BZ was responsible for theory development, writing, and framing of this manuscript. AElse supervised the dissertation completion of the AElsa. AG supervised the first author’s literature review, data collection for his dissertation work, and contributed in a concrete way in the revision and resubmission process. All authors contributed to the article and approved the submitted version.
